# Icosapent Ethyl (Vascepa®) for the Treatment of Acute, Severe Pancreatitis

**DOI:** 10.7759/cureus.11551

**Published:** 2020-11-18

**Authors:** Amnon A Berger, Robert Sherburne, Ivan Urits, Haresh Patel, Jonathan Eskander

**Affiliations:** 1 Department of Anesthesia, Critical Care, and Pain Medicine, Beth Israel Deaconess Medical Center, Harvard Medical School, Boston, USA; 2 Critical Care Medicine, Maryview Medical Center, Portsmouth, USA; 3 Anesthesiology and Pain Medicine, Portsmouth Anesthesia Associates, Portsmouth, USA

**Keywords:** covid 19, epa, omega-3, inflamation, cytokine, cytokine release syndrome (crs), intensive respiratory care, critical care and hospital medicine

## Abstract

Acute pancreatitis is the most common gastrointestinal pathology that warrants hospital admission, with an estimated incidence of 13-45/100,000 annually in the US. The overall mortality is low but is significantly increased in 15-25% of patients that develop severe disease, likely secondary to an increase in inflammation and an exaggerated response, sometimes referred to as a cytokine storm. Management is largely supportive, and no specific cure exists to hasten recovery.

Icosapent Ethyl (IPE, Vascepa®) is an omega-3 fatty acid derivative that is indicated for the treatment of hypertriglyceridemia and has been shown to improve mortality from cardiovascular causes, likely through an anti-inflammatory mechanism. We report here a case of very severe, abrupt acute alcoholic pancreatitis in a 31-year-old male, requiring intensive care unit admission, ventilation, and support with multiple vasoactive medications. Shortly after the initiation of IPE, the patient started to improve and ultimately made a complete recovery. His initially greatly elevated inflammatory markers downtrended quickly under IPE treatment and he followed with a remarkable clinical recovery.

Several previous studies, such as the Patients With Persistent High Triglyceride Levels (≥ 200 mg/dL and < 500 mg/dL) Despite Statin Therapy (ANCHOR; NCT01047501) and the Multi-Center, PlAcebo-Controlled, Randomized, Double-BlINd, 12-week study with an open-label Extension (MARINE; NCT01047683), provided evidence of the anti-inflammatory activity of IPE. In our case, we provide the first evidence to support its use as a direct anti-inflammatory in severe disease. With the absence of direct therapy and the significant mortality from severe acute pancreatitis, IPE can be a breakthrough therapy. Its treatment is not limited to pancreatitis only, and it may also be beneficial in other cases of severe inflammation. Though anecdotal, this case provides evidence to support further study of IPE in states of exaggerated inflammation.

## Introduction

Pancreatitis is the inflammation of the pancreas and is diagnosed with the existence of two of the following three findings: persistent epigastric pain that radiates to the back, a three-fold increase in serum lipase or amylase, and imaging findings that are consistent with pancreatitis [1]. Acute pancreatitis (AP) is the most frequent gastrointestinal cause for hospital admission, and it occurs in an estimated 13-45/100,000 persons in the US. In 2009 alone, 275,000 cases were reported [2]. Gallstones are the single most common cause for AP, with alcohol consumption the second most common risk factor. Two to five percent of chronic drinkers will suffer from AP [2]. Alcohol is responsible for approximately 25-35% of the cases of AP in the US. The other common causes include hypertriglyceridemia, post-endoscopic retrograde cholangiopancreatography (ERCP), genetic risks, drug-induced, hyperparathyroidism, and pancreatic duct injury [3].

The overall mortality from AP is about 5%, with a lower probability for the majority of patients (85%) with interstitial edematous pancreatitis; however, up to 15% of cases will experience necrosis of the pancreas, with an associated mortality of about 17%. Complications are mostly local in origin; however, exacerbation of underlying disease and multiorgan system failures are less common, severe manifestations [1]. Though the overall mortality from AP is decreasing, 15-25% of patients develop severe AP, defined as persistent organ failure for more than 48 hours, and is associated with higher morbidity and mortality. Older age, alcoholic pancreatitis, short time to symptom onset, and obesity have all been described as risk factors for severe AP. Early and persistent organ failure is a reliable indicator for both prolonged hospital stay as well as increased mortality. The sequential organ failure assessment (SOFA) algorithm attempts to score organ failure, with increasing numbers predicting higher mortality. Other scores, such as Ranson’s criteria, Acute Physiology and Chronic Health Evaluation (APACHE II), and Systemic Inflammatory Response Syndrome (SIRS) also attempt to predict the development of significant disease. The development of severe disease is likely related to increased inflammation and a cytokine storm, evident by elevated serum levels of inflammatory markers [4].

The management of AP is largely supportive and consists primarily of hydration and pain control. Most patients will be able to tolerate oral intake soon after diagnosis, and in those with more severe disease, nutrition must also be evaluated; oral feeding is preferred when postprandial pain is not too severe, and feeding is not limited by local complications. When oral feeding is not possible, enteral feeding should be preferred over parenteral nutrition; the latter should be reserved for patients not tolerating enteral feeding. Antibiotics are reserved for either infected necrotizing pancreatitis or extrapancreatic infection and should not be administered prophylactically. The management of complications is mostly reserved for local difficulties, and no specific treatment targets inflammation in severe disease. Treatment of risk factors, such as lipid-lowering medications and alcohol abstinence, follows acute disease to reduce the risk of recurrence. Chronic pancreatitis will ultimately develop in about 10-36% of patients with AP [1].

Ethyl eicosapentaenoic acid or icosapent ethyl (IPE) is marketed as Vascepa® and indicated for the treatment of persistent hypertriglyceridemia. It is a pure prescription form of eicosapentaenoic acid (EPA) ethyl ester, an omega-3 fatty acid derivative that is indicated for persistent hypertriglyceridemia as an adjunct to maximally tolerated statin therapy and proven to reduce lipid levels, as well as cardiovascular risk [5,6]. The mechanism in which IPE reduced cardiac risk in the Reduction of Cardiovascular Events with Icosapent Ethyl - Intervention Trial (REDUCE-IT; NCT1492361) is less understood given that the triglyceride-lowering effect of IPE was modest at best. Two suggested mechanisms include anti-platelets and anti-inflammatory effects. The latter is supported by evidence showing a significant reduction in inflammatory markers with the use of IPE in both the ANCHOR and MARINE trials [7-9]. We recently published our successful experience in treating a case of coronavirus disease 2019 (COVID-19) with IPE [10], further contributing to this hypothesis; a treatment that is now tested in ongoing clinical trials [11]. IPE’s use is currently limited to the above-described indication, and it is not used as an anti-inflammatory drug. Here, we report the case of a patient with severe alcoholic AP successfully treated with IPE.

## Case presentation

**Figure 1:**
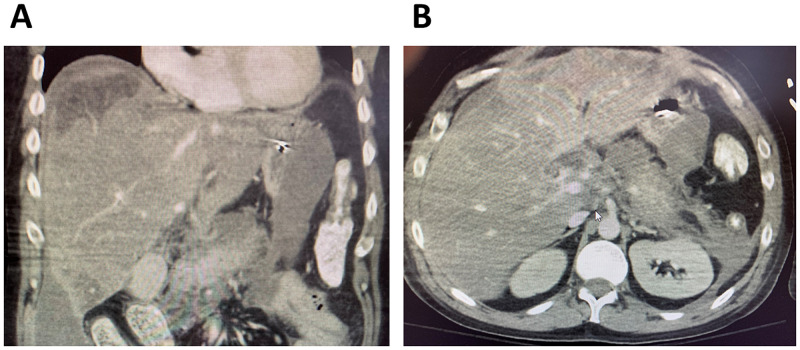
Abdominal CT demonstrating acute pancreatitis The patient presented with abdominal pain and elevated lipase, concerning for acute pancreatitis. An abdominal CT (A: sagittal, B: axial) scan was obtained, demonstrating extensive soft tissue and fat stranding about the pancreas and adjacent to the peritoneum, confirming the diagnosis. Given his extensive drinking history, a diagnosis of alcoholic pancreatitis was made.

A 31-year-old man with a past medical history of heavy alcohol use for the past 14 years presented to the hospital with nausea and epigastric pain radiating to his back for one day. A diagnosis of pancreatitis was made with a classical presentation of abdominal pain and serum lipase elevated to 3184 U/L. The diagnosis was confirmed with an abdominal CT scan showing extensive soft-tissue stranding about the pancreas and adjacent duodenum (Figure 1). A right upper quadrant ultrasound ruled out gallstone pancreatitis or ductal dilation. The patient was found to have a Bedside Index for Severity in Acute Pancreatitis (BISAP) score of 3 and admitted to the medicine floor. The patient was given IV hydration with lactated ringer (LR), opioid analgesics, and antiemetic medication. His alcohol withdrawal symptoms were treated with a single dose of chlordiazepoxide and as-needed lorazepam per his clinical institute withdrawal assessment (CIWA) score.

On his second day of admission, approximately 30 hours after presentation, the patient was found to have altered mental status. Arterial blood gasses drawn during a rapid response to his clinical deterioration indicated he was in severe metabolic acidosis with inadequate respiratory compensation with a pH of 6.857, pCO2 48 mmHg, pO2 100 mmHg, and calculated HCO3- of 8.5 mmol/L. His potassium was 6.7 mmol/L, creatinine 5.3 mg/dL, calcium 5.6 mg/dL, lipase 11,000 U/L, aspartate aminotransferase (AST) 12800 U/L, alanine aminotransferase (ALT) 1759 U/L, up from 275 U/L and 256 U/L, respectively, at presentation, lactic acid 5.4 mg/dL, and total creatine kinase (CK) was 5000 U/L. He was transferred to the intensive care unit (ICU) and rapidly given 3L of intravenous fluid (IVF) with normal saline (NS), intubated for airway protection and respiratory support, started on a bicarbonate drip, initiated on continuous renal replacement therapy (CRRT). He was also started on an IV infusion of norepinephrine, vasopressin, and neo-synephrine (phenylephrine) to maintain adequate mean arterial pressure (MAP). Blood cultures were drawn, and empiric antibiotic treatment with vancomycin and piperacillin-tazobactam (Pip-Tazo) was initiated for suspected sepsis. His SOFA score was calculated to be 18, indicating mortality from AP in excess of 95%.

**Figure 2:**
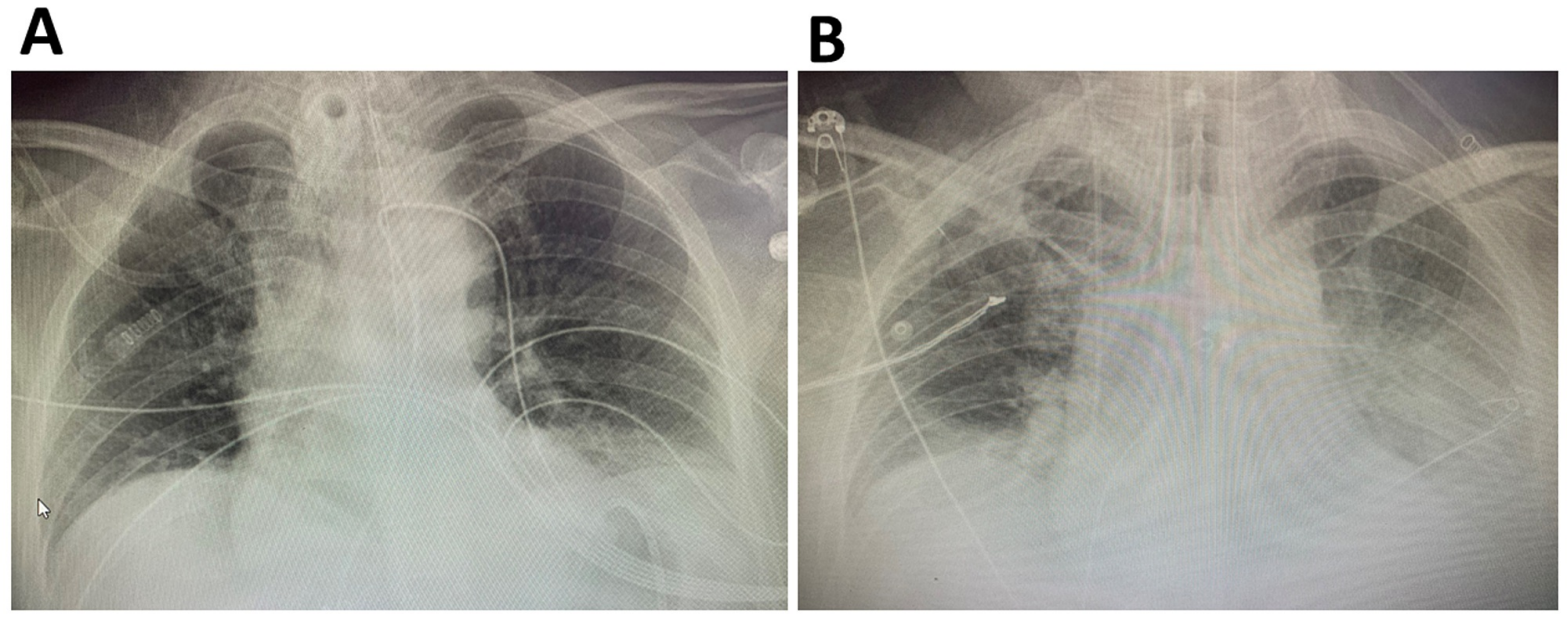
Chest X-rays before and after IPE initiation Chest X-rays demonstrating pulmonary status prior (A) and two weeks after (B) IPE initiation.

After 24 hours of ICU admission, the patient showed little clinical improvement. Though his serum lipase level trended down to 5900 U/L, his hepatocellular enzymes were continuing to increase (AST 14100 U/L, ALT 1800 U/L), likely secondary to shock and hepatic hypoperfusion; his total CK increased to 12700 U/L, and he still required three vasoactive IV infusions to maintain MAP. Furthermore, his temperature increased to 38.1C. Given the COVID-19 epidemic occurring during this hospitalization, and previous reports of COVID-19 related pancreatitis [12,13], a severe acute respiratory syndrome coronavirus 2 (SARS-CoV-2) swab was also sent, however, his SARS-CoV-2 testing ultimately returned negative, as well as the original and repeat blood cultures. A chest X-ray also showed no sign of infection, and there were no other signs of extrapancreatic infection (Figure 2).

During his ICU stay, a gastric feeding tube (G-Tube) was placed, and he was given enteral feeding. By hospital day three, he was still showing little clinical improvement, and with sterile cultures and no indication of active infection, his diagnosis was likely that of cytokine storm and increased inflammation rather than active infection, driving his severe immune response syndrome (SIRS) and distributive shock. He was started on a 4g daily IPE dose (2g twice daily) via G-tube. Following this administration, he began to improve; by the next morning, his bicarbonate infusion had been turned off, and later that day, he was weaned off of neo-synephrine. Within 24 hours of IPE administration, lab results were improved for all indices: lipase went down to 2458 U/L, AST 3918 U/L, ALT 933 U/L, and total CK 8100 U/L. By admission day five, he had also weaned off norepinephrine, and by day six required no vasoactive medications as vasopressin was discontinued. Inflammatory markers continued to decrease; however, during hospital day six, he developed a recurrent fever, and a CT demonstrated an interval development of lower lobe lung consolidation, concerning for pneumonia. The patient continued to improve despite this, CRRT was exchanged to intermittent hemodialysis (HD). Despite an initially increasing leukocytosis and continued fevers, attributed to pneumonia, the patient ultimately was discharged from the ICU for continued care on the medicine floor. His total stay in the hospital was approximately four weeks, but returned home in good health.

## Discussion

In the present clinical presentation, we have described a case of a 31-year-old patient with chronic ethanol abuse who presented with severe AP and survived against overwhelming odds and a protracted, complicated hospital course. Given his apparent sterile pancreatitis and likely cytokine storm, he was treated with IPE for its anti-inflammatory effects.

Omega-3 derivatives and EPA have previously been studied in inflammation, SIRS, and sepsis, and their mechanism of action is likely multi-faceted. Omega-3 fatty acids and their derivatives were previously shown to have antimicrobial effects and inhibit the growth of bacteria and were shown to have antiviral activity, including against herpes and influenza species [14]. Though in our case, the patient had no known active infection at first, this may have had an effect on preventing one from developing, as well as attenuating his pneumonia course. In vitro studies have also demonstrated the efficacy of EPA in endothelial cell stabilization and improvement of function, which is likely also important in a distributive shock [15,16].

The most likely mechanism of action of IPE, in this case, is the attributed anti-inflammatory role as well as cell membrane stabilization. Omega-3 and EPA were previously investigated in that perspective, and consistent results showed an in vitro reduction of inflammatory cytokines, specifically tumor necrosis factor α (TNFα) and nuclear factor kappa B (NF-κB) [17,18]. It is very likely that the cardiovascular protection attributed to IPE in the REDUCE-IT trial is secondary to the reduction of inflammation, given the growing body of evidence suggesting that atherosclerosis is a chronic inflammatory process, and especially in light of the only modest reduction in lipid levels in the intervention group [19]. Interestingly, only IPE, the pure ethyl-ester form, was found to be effective in this trial [6], which is likely due to the differences between in vitro systems and in vivo conditions.

Indeed, analysis of results from the ANCHOR and MARINE trials studying IPE in different populations persistently demonstrated a significant reduction in inflammatory markers with IPE use. Most notably, high-sensitivity C-reactive protein (hsCRP), a sensitive, nonspecific marker of inflammation, was shown to decrease with IPE treatment [7-9,20]. These studies support the hypothesis that IPE has anti-inflammatory properties.

## Conclusions

In our case, our patient with likely cytokine storm improved clinically almost immediately after the administration of IPE, despite poor prognosis and lacking response to previous therapeutic attempts. This suggests that the reduction of inflammation likely participated in his clinical improvement. One could also hypothesize that direct anti-hypertriglyceridemic treatment may have been beneficial for AP in this case.

Further research is required to explore the efficacy of IPE in SIRS and AP. To our knowledge, outside of in vitro studies, this is the first documentation of IPE used for cytokine release storm. It is also the first description of IPE use for treatment of AP. Larger clinical studies will be required to elucidate the efficacy of IPE in these situations. Our description will hopefully trigger larger case series and clinical studies.

## References

[1] (2019). Clinical Manifestations and Diagnosis of Acute Pancreatitis. https://www.uptodate.com/contents/clinical-manifestations-and-diagnosis-of-acute-pancreatitis.

[2] Yadav D, Lowenfels AB (2013). The epidemiology of pancreatitis and pancreatic cancer. Gastroenterology.

[3] (2019). Etiology of Acute Pancreatitis. https://www.uptodate.com/contents/etiology-of-acute-pancreatitis.

[4] (2019). Predicting the Severity of Acute Pancreatitis. https://www.uptodate.com/contents/predicting-the-severity-of-acute-pancreatitis.

[5] Arnold SV, Bhatt DL, Barsness GW (2020). Clinical management of stable coronary artery disease in patients with type 2 diabetes mellitus: a scientific statement from the American Heart Association. Circulation.

[6] Bhatt DL, Steg PG, Miller M (2019). Cardiovascular risk reduction with icosapent ethyl for hypertriglyceridemia. N Engl J Med.

[7] Miller M, Ballantyne CM, Bays HE (2019). Effects of icosapent ethyl (eicosapentaenoic acid ethyl ester) on atherogenic lipid/Lipoprotein, apolipoprotein, and inflammatory parameters in patients with elevated high-sensitivity C-reactive protein (from the ANCHOR Study). Am J Cardiol.

[8] Brinton EA, Ballantyne CM, Bays HE (2013). Effects of icosapent ethyl on lipid and inflammatory parameters in patients with diabetes mellitus-2, residual elevated triglycerides (200-500 mg/dL), and on statin therapy at LDL-C goal: The ANCHOR study. Cardiovasc Diabetol.

[9] Bays HE, Ballantyne CM, Braeckman RA (2015). Icosapent ethyl (eicosapentaenoic acid ethyl ester): effects upon high-sensitivity C-Reactive protein and lipid parameters in patients with metabolic syndrome. Metab Syndr Relat Disord.

[10] Berger AA, Sherburne R, Urits I, Patel H, Eskander J (2020). Icosapent ethyl - a successful treatment for symptomatic COVID-19 infection. Cureus.

[11] (2020). EPA-FFA to Treat Hospitalised Patients With COVID-19 (SARS-CoV-2). https://clinicaltrials.gov/ct2/show/NCT04335032.

[12] Hadi A, Werge M, Kristiansen KT (2020). Coronavirus Disease-19 (COVID-19) associated with severe acute pancreatitis: case report on three family members. Pancreatology.

[13] Anand ER, Major C, Pickering O, Nelson M (2020). Acute pancreatitis in a COVID-19 patient. Br J Surg.

[14] Das UN (2006). Do unsaturated fatty acids function as endogenous antibacterial and antiviral molecules?. Am J Clin Nutr.

[15] Mason RP, Dawoud H, Jacob RF, Sherratt SC, Malinski T (2018). Eicosapentaenoic acid improves endothelial function and nitric oxide bioavailability in a manner that is enhanced in combination with a statin. Biomed Pharmacother.

[16] Lee CH, Lee S Da, Ou HC, Lai SC, Cheng YJ (2014). Eicosapentaenoic acid protects against palmitic acid-induced endothelial dysfunction via activation of the AMPK/eNOS pathway. Int J Mol Sci.

[17] Zhao Y, Joshi-Barve S, Barve S, Chen LH (2004). Eicosapentaenoic acid prevents LPS-induced TNF-α expression by preventing NF-κB activation. J Am Coll Nutr.

[18] Serini S, Bizzarro A, Piccioni E (2012). EPA and DHA differentially affect in vitro inflammatory cytokine release by peripheral blood mononuclear cells from Alzheimer’s patients. Curr Alzheimer Res.

[19] Harris WS (2019). Understanding why REDUCE-IT was positive - mechanistic overview of eicosapentaenoic acid. Cardiovasc Dis.

[20] Bays HE, Ballantyne CM, Braeckman RA, Stirtan WG, Soni PN (2013). Icosapent ethyl, a pure ethyl ester of eicosapentaenoic acid: effects on circulating markers of inflammation from the MARINE and ANCHOR studies. Am J Cardiovasc Drugs.

